# Linear filtering reveals false negatives in species interaction data

**DOI:** 10.1038/srep45908

**Published:** 2017-04-06

**Authors:** Michiel Stock, Timothée Poisot, Willem Waegeman, Bernard De Baets

**Affiliations:** 1 KERMIT, Department of Mathematical Modelling, Statistics and Bioinformatics, Ghent University, Coupure links 653, Ghent B-9000, Belgium; 2 Université de Montréal, Département des Sciences Biologiques, 90 Avenue Vindent d’Indy, Montréal, QC, H2V 3S9, Canada

## Abstract

Species interaction datasets, often represented as sparse matrices, are usually collected through observation studies targeted at identifying species interactions. Due to the extensive required sampling effort, species interaction datasets usually contain many false negatives, often leading to bias in derived descriptors. We show that a simple linear filter can be used to detect false negatives by scoring interactions based on the structure of the interaction matrices. On 180 different datasets of various sizes, sparsities and ecological interaction types, we found that on average in about 75% of the cases, a false negative interaction got a higher score than a true negative interaction. Furthermore, we show that this filter is very robust, even when the interaction matrix contains a very large number of false negatives. Our results demonstrate that unobserved interactions can be detected in species interaction datasets, even without resorting to information about the species involved.

Biological data such as microscopy images, environmental sensor readings and species incidence counts are inherently noisy. Often a simple linear transformation can be applied to obtain a denoized re-estimation of the data^1^. For instance, a noisy image can be rectified by applying a filter that exploits the fact that adjacent pixels in an image tend to have similar values^2^. Similarly, species interaction values are not randomly distributed, but exhibit structures such as nestedness^3^,^4^, modularity^5^ or low-dimensional embedding^6^. Since these interactions are largely determined by evolved traits of both partners^7^,^8^,^9^, a filter for these types of data could take this information into account.

Machine learning methods, often based on kernels, have been applied with great success in similar cases, for example to predict interaction values between biomolecules based on sequence information^10^,^11^,^12^, but seem to have remained absent from an ecological context. If no side information such as traits or phylogeny of the individual species is available, only the structure of the interaction dataset can be exploited. This can be realized by letting the filtered interaction values not only depend on the observed interaction, but also on the degree to which the two species in the interactions are involved in other interactions. Let *Y* = [*Y*_*ij*_] be the sparse *n* × *m* matrix of interaction values, either a binary matrix or a matrix of positive real numbers expressing interaction strength. We refer to the non-zero values, i.e. detected interactions, as positive interactions, and to the zero values, i.e. absent interactions, as negative interactions. In ecological literature, ‘positive interaction’ is often used to refer to an interaction in which both species benefit (e.g. symbiosis), while ‘negative interaction’ is used for an interaction where one of the species has a disadvantage (e.g. parasitism). In this work, we use the term positive (resp. negative) interactions to refer to an observed (resp. unobserved) interaction, regardless of the nature of the interaction. This is more consistent with standard statistical terminology.

The filtered interaction matrix *F* = [*F*_*ij*_] can be obtained as the following weighted average of averages:


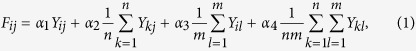


where 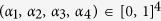 and 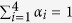. The first term is proportional to the interaction value, while the last term is proportional to the average of all interaction values in the matrix. The second (resp. third) term is proportional to the average of the values in the corresponding column (resp. row), i.e. relative to the promiscuity of the individual species. The parameters *α*_1_, *α*_2_, *α*_3_ and *α*_4_ act as weighting coëfficiënts. This filter is illustrated on a toy dataset in Fig. 1(a–c).

Usually, interaction datasets are sampled by monitoring one of the species types and observing the number of interactions with the species of the other type^13^ (e.g. studying the fecal matter of predators to assess their preys or keeping track of pollinators landing on plants). As a consequence, these interaction matrices are often undersampled and some zeros might be false negatives rather than true negative interactions^14^,^15^. This can lead to some serious biases in descriptors derived from such matrices^13^,^16^,^17^,^18^. To assess whether a particular interaction between species *i* and species *j* is likely to occur in reality according to the dataset, one should ideally not make use of the observed interaction value *Y*_*ij*_. We therefore impute this interaction value, further on denoted as *β*, in such a way that when it is passed through the filter, it remains unchanged. This embodies the rationale that we want to impute the interaction value to closely match the rest of the data according to the filter. Consider Eq. (1) using a copy of *Y* where *Y*_*ij*_ is replaced by *β*, then it should hold that:


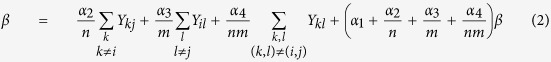



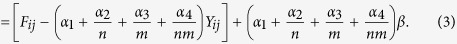


This is illustrated in Fig. 1(d–f) for the toy dataset. Solving for *β*, we obtain


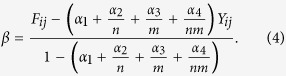


This imputation does *not* depend on the original value of *Y*_*ij*_, as can be gleaned from Eq. (2). Only the other interaction values in the dataset contribute to the imputation. The process of imputing the interaction values one by one is known as leave-one-out (LOO) imputation. Equation (4) is a special case of the well-known LOO shortcut^19^ and provides a computationally efficient way of performing LOO imputation.

As a simple method to detect false negatives in interaction matrices, we suggest to score negative interactions in datasets using LOO imputation and rank the negative interactions according to this score. The last term in Eq. (1), i.e. the average interaction value, will not influence the ranking of interactions. However, if the goal is to impute the interaction value to some degree of accuracy, this term provides an essential contribution. Negative interactions that receive high scores during imputation are potential false negatives and should be closer examined. In the experiments we will demonstrate, first, that imputations of positive interactions will on average result in higher scores than negative interactions and, second, that false negatives in turn receive higher scores than true negatives, making this a suitable method for false negative discovery. The proposed linear filter will be compared to the use of a low-rank approximation of the interaction matrix, obtained through singular value decomposition (SVD), a popular method to impute missing values in collaborative filtering^20^,^21^. The re-estimation using SVD is obtained by retaining only the leading eigenvalues of the matrix *Y* after decomposition. Since the eigenvalue spectrum of the interaction dataset is related to the nestedness of the network^22^, it seems sensible that this method could work well for nested interaction networks. Our filter works demonstratively better than SVD in most cases and remains performant even with very high rates of false negative interactions. Finally, we illustrate that when forbidden links (i.e. true negatives) are known, the performance can be increased slightly.

## Material and Methods

In our experiments we used a series of species interaction datasets obtained from the Interaction Web DataBase (https://www.nceas.ucsb.edu/interactionweb/resources.html) and Web of Life database (http://www.web-of-life.es/). We only withheld datasets with at least ten rows and ten columns, leaving us with 180 datasets describing anemone-fish, host-parasite, plant-ant, plant-herbivore, plant-seed dispensers, plant-pollinator and predatory-prey interactions. We have chosen such a diverse catalogue of datasets to illustrate that the proposed method is broadly applicable. Some datasets contained only binary absence-presence information, others contained valued interactions, such as frequency of visits. Our method can be applied regardless. All datasets were quite sparse, with an average positive interaction density *ρ* of 0.15 ± 0.12 (average value ± standard deviation calculated over the different datasets).

In this work we investigate whether the scores of imputed interaction values can be used to discriminate between unobserved positive and negative interactions. As a performance metric, we will use the area under the ROC curve (AUC), calculated as


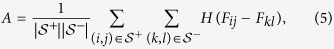


with *F*_*ij*_ the imputed score, 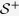 (resp. 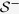) the set of the positive (resp. negative) interactions and *H*(·) the Heaviside step function. The AUC can be interpreted as the probability that a randomly chosen positive interaction receives a higher score than a randomly chosen negative interaction.

The LOO imputations of the interaction datasets were computed using Eq. (4). Since we use AUC to evaluate the imputations, we are not interested in the exact values. Rather, positive interactions should on average receive higher imputed values compared to negative interactions. A small explorative study on a couple of datasets has shown that our ranking-based evaluation using AUC is quite insensitive to the exact values of the parameters of the filter. Hence, we have set all parameters equal, i.e. (*α*_1_, *α*_2_, *α*_3_, *α*_4_) = (0.25, 0.25, 0.25, 0.25), meaning that each of the four averages in Eq. (1) has the same weight. The filter is thus reduced to a standard average. If the filter would be used to estimate the probability of interaction or the interaction strength, we recommend to do some tuning of the parameters to the dataset at hand, for example, using cross-validation to minimize squared loss.

## Results

First, we show that a positive interaction receives a higher score than a negative interaction. For each dataset, we calculated the LOO imputation and compared the scores of the positive and the negative interactions. The average AUC was found to be 0.77 ± 0.10, meaning that on average there is about 77% chance that a missing positive interaction will receive a higher score than a missing negative interaction. Intriguingly, we found that using the strength of the interactions tends to decrease the performance. When datasets containing strength of interactions were binarized by setting positive values to one, the performance increased on average with 3.5% ± 4.4%. A paired *t*-test showed that this increase in average AUC is significant at the 0.01 level (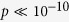, *n* = 94 datasets). This implies that in many cases the strength of interaction is too noisy to be exploited by the filter. This was to be expected, as quantitative interaction strength depends on local conditions^23^,^24^, and is therefore more susceptible to noise. Hence, making the interaction matrix binary often leads to more robust filtering.

**Figure 2:**
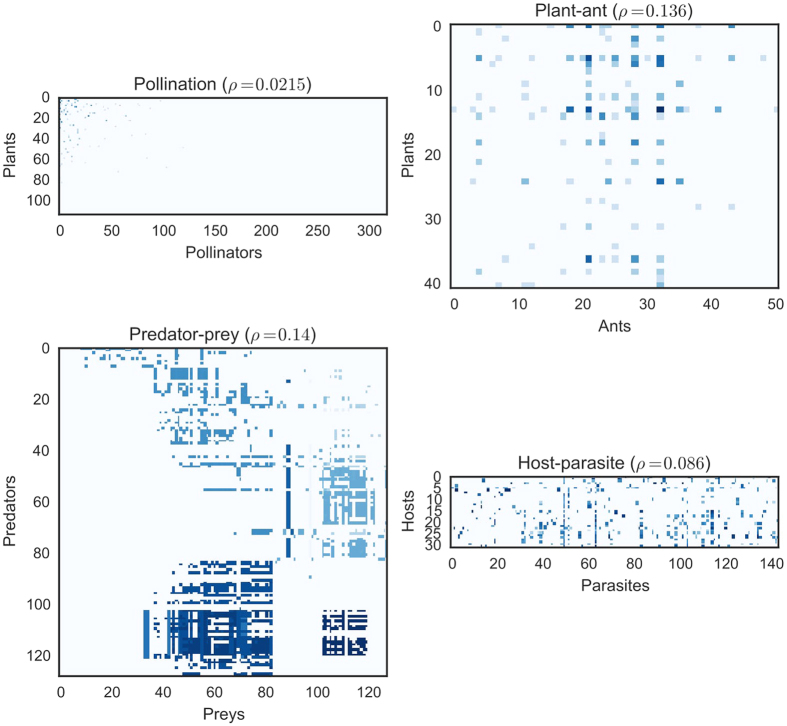
Heat maps of four valued species interaction datasets with the corresponding density *ρ*. The brightness of the color corresponds to the value of the interaction.

**Figure 3:**
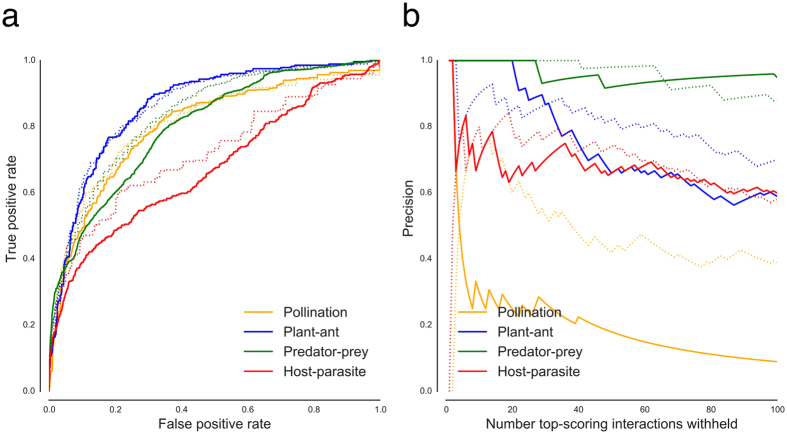
Results of the imputation experiments using the four datasets shown in Fig. 2. (**a**) ROC curves for the scores of the LOO imputation. (**b**) The precision of detecting true interactions as a function of the size of top-scoring interactions. In both plots full lines represent experiments where the intensity of the interactions was used and broken lines represent experiments where the interaction dataset was binarized.

Four sizeable datasets representing different types of interactions^25^,^26^,^27^,^28^,^29^ were studied in more detail, see Fig. 2. In Fig. 3(a) the ROC curves illustrate that usually a large fraction of the positive interactions can easily be detected without obtaining many false positives. This is important for practical applications, as these high-scoring interactions should be used to decide which interactions are promising for validation in the field. The top-scoring interactions are strongly enriched with positives, as illustrated in Fig. 3(b), which shows the precision (fraction of top-scoring positive interactions) as a function of the size of the top. Although the individual patterns vary with the density, distribution and sampling effort of the interaction datasets, here one can observe also a clear trend that making the datasets binary results in higher precision. On average, for all datasets, the precision at the top-10 was 0.69 ± 0.27, which is substantially higher than the average density of 15%, the expected precision of a random scoring.

**Figure 4:**
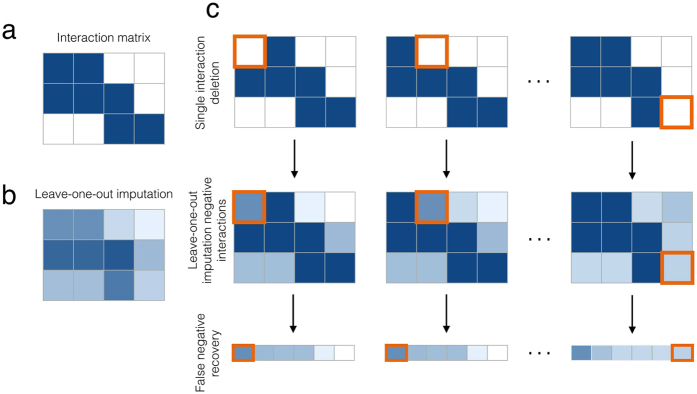
(**a**) A small binary interaction matrix *Y* of three by four species. (**b**) The corresponding matrix where each value is computed by LOO imputation. The score of each interaction is calculated only based on the values of all other interactions, without its original value. (**c**) To test if a false negative can be detected, each positive interaction is made negative one by one, indicated in orange. For each of these changed datasets, all negative interactions are scored using loo imputation. The negative interactions are sorted by their scores and the position of the false negative, indicated by an orange frame, is determined.

Since most species interaction datasets are obtained through observation studies, negative interactions may either indicate that the species do not interact in practice or that their interaction is not observed during the study. To show that linear filtering can reveal false negatives, we created variants of each dataset, each with exactly one positive interaction made negative, and did this for every positive interaction. Subsequently, all negative interactions were scored using LOO imputation and the score of the false negative was compared with the scores of the true negatives (Fig. 4). The average AUC for detecting these false negatives was 0.78 ± 0.098, averaged over all the 180 datasets. Again, when the interaction datasets containing strength of interaction were binarized, the performance increased with on average 4.0% ± 4.4%. Using a paired *t*-test, this increase in average AUC was also found to be significant at the 0.01 level (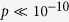, *n* = 94 datasets). Whereas the previous experiment showed that positive interactions receive higher scores than negative interactions, this experiment demonstrates that within the negative interactions, false negatives tend to receive higher scores than true negatives. Table 1 summarizes the AUC scores obtained for the two described experiments.

Even when many interactions are missing, our method remains performant. In an additional experiment, first, we illustrate how the performance of the linear filter changes with larger fractions of false negatives and, second, we compare the linear filter to the use of a low-rank approximation of the interaction matrix *Y* obtained by SVD. SVD can be used to obtain the closest approximation in terms of mean squared error of a matrix for a given rank. The rank was chosen as the lowest rank such that the approximated dataset retained at least 75% of the variance of the original dataset. The re-estimated matrix was evaluated the same way as the matrix obtained by LOO imputation using the linear filter. Experiments using both the linear filter and the SVD approximation were performed on the four datasets in Fig. 2, by randomly setting 5%, 10%, 20%, 50% or 90% of the positive interaction values to zero. Using AUC, we assessed how well the re-estimated interaction values could be used to discriminate between true and false negatives. Re-estimation was done using both the original interaction datasets and versions of the datasets where the interaction values were binarized. Each experiment was repeated 100 times. The performances are listed in Table 2. For three datasets, the linear filter clearly shows a better performance. Interestingly, SVD seems to work really well on the predator-prey dataset, a large dataset with visually a strong structural pattern. Nevertheless, using the linear filter usually leads to a good performance, especially since most interaction matrices are rather small. This filter also seems to be still able to detect false negative interactions even when the percentage of false negatives is very high, in contrast to using the low-rank approximation. This indicates that our method is quite robust, even when the datasets contain many missing values.

**Figure 5:**
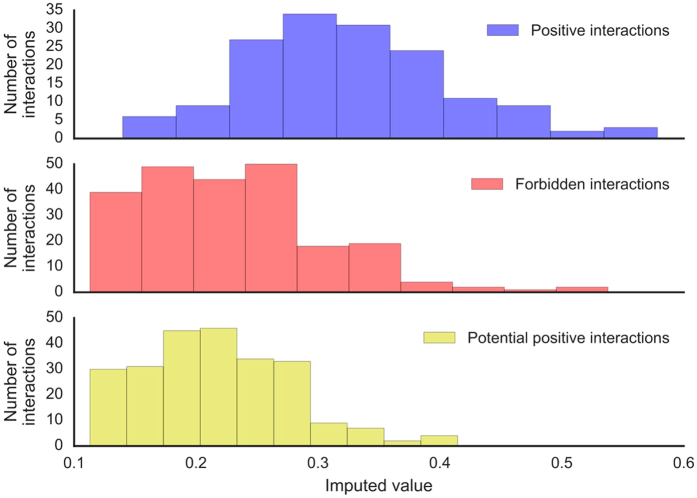
Histogram of the imputed values for the positive interactions, forbidden interactions and negative interactions, which are potential false positives. The positive interactions are on average imputed with a higher score than both kinds of negative interactions.

Finally, we performed a small experiment where true negatives or forbidden links are known. To this end, we use the 25-by-25 seed-dispersal network of Olesen and coauthors^30^. It consists of 156 observed positive interactions and 228 forbidden interactions due to phenological uncoupling or morphological constraints. We used the linear filter to perform LOO imputation on the interaction matrix. Figure 5 shows the distributions of the imputed values for the positive interactions, true negative interactions and negative interactions that are potential false positives. The AUC for discriminating between positive and negative interactions (both true negatives and false negatives) using LOO imputation was found to be 0.8270. When only trying to discriminate between true positives and true negatives, the AUC was 0.7981. Upon removing the true negatives, the AUC improved slightly to 0.8543. For this dataset, it seems that the true negatives are somewhat harder to identify than the negatives in general. When true negatives are known, it is best to only search for false negatives within the potentially positive interactions.

## Discussion

Evidently, the latent information in the interaction matrices can be used to detect unobserved (false negative) interactions. We are convinced that techniques such as linear filtering may allow to either directly ameliorate an interaction dataset or can be used to suggest promising interactions that can subsequently be verified in the field. Making use of *in silico* predicted interaction scores to suggest experiments *in vitro* is already commonplace in domains such as drug discovery^31^ and can be seen as part of the broader paradigm of recommender systems^32^,^33^. Negative interactions with high scores are natural targets for increased sampling effort, as they are most likely to occur in reality.

Standard algorithms for recommender systems make recommendations by exploiting structures in the data, e.g. low-rankness of the interaction matrix^34^. This idea could be applied to predict the value of missing interactions. For example, it has been used successfully to predict the joint growth between heterotrophic and methanotrophic bacteria^35^. Other methods for filtering a network could be based on different principles, for example the stochastic block model^36^. In essence, the simple linear filter of Eq. (1) and the associated imputation formula (4) only use information on row and column counts to do an imputation. We can motivate the use of this filter in three ways. Firstly, it is a very simple first method to try to infer false negatives. Although despite having four parameters, their exact value is less important if one is only interested in ranking interactions, so not much tuning is required. Secondly, the filter is very robust and works demonstratively well on small datasets and with a very large fraction of false negatives. Finally, using the shortcut for LOO cross validation, it is very easy and computationally efficient to get a realistic estimate of the performance of the filter for a given dataset. More complex methods are expected to yield better performance, but require to be tuned more carefully to the dataset at hand.

Often, one has information about the individual species, such as geographical location, morphology or phylogeny, which can also be incorporated to predict interaction^8^,^37^,^38^. Using such side information, denoted as content-based filtering in recommender systems^32^, can improve the accuracy of the prediction as well as explain the interactions based on species traits, if used in combination with model selection tools. As we have not incorporated such information in our method, the performances presented in this work can be seen as a lower bound for detecting missing interactions.

## Additional Information

**How to cite this article:** Stock, M. *et al*. Linear filtering reveals false negatives in species interaction data. *Sci. Rep.* **7**, 45908; doi: 10.1038/srep45908 (2017).

**Publisher's note:** Springer Nature remains neutral with regard to jurisdictional claims in published maps and institutional affiliations.

## Figures and Tables

**Figure 1:**
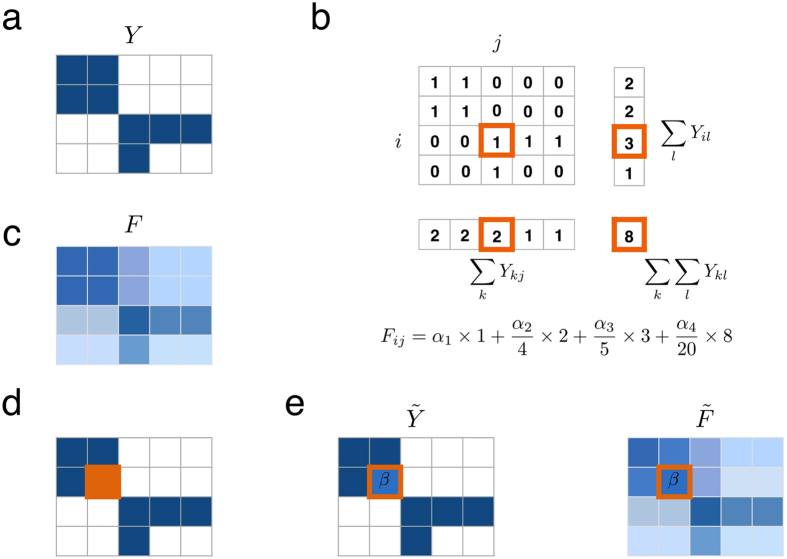
(**a**) A small binary interaction dataset *Y* of four by five species. (**b**) Example of how to calculate the filtered value for *Y*_*ij*_. The filter in Eq. (1) computes a weighted average of the observed value itself, the column and row averages and the average of all interaction values in the matrix. (**c**) The filtered dataset *F* corresponding to *Y*. (**d**) The dataset *Y* where one value is to be imputed, indicated in orange. The imputation of this interaction value should be independent of the original, possibly wrong, value. (**e**,**f**) The value *β* of the imputed interaction does, by definition, not change when passing through the filter.

**Table 1:** Average AUC, aggregated for different densities *ρ* and different total numbers of positive interactions in all the different datasets.

Density *ρ*	Imputation AUC			False negative recovery AUC		
	[0, 0.1]	[0.1, 0.25]	[0.25, 1]	[0, 0.1]	[0.1, 0.25]	[0.25, 1]
#Interactions						
[0, 50]	0.8187	0.6119	0.7180	0.8443	0.6489	0.7426
[50, 100]	0.7561	0.7194	0.8017	0.7696	0.7341	0.8190
[100, 1000]	0.8259	0.7857	0.8002	0.8301	0.7925	0.8088
[1000, 10000]	0.8219	0.8423	—	0.8232	0.8429	—
[10000, +∞]	0.8482	—	—	0.8486	—	—

The first part gives the results for the imputation experiments, the second part presents the results for the false negative recovery experiments. All datasets with interaction strengths were binarized for these experiments.

**Table 2:**
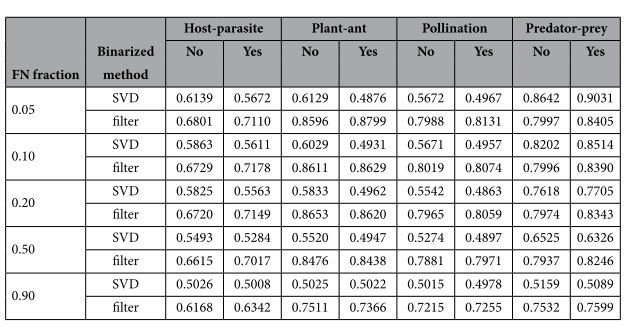
Comparison of the linear filter with SVD for an increasing fraction of randomly assigned false negatives (FN) for four datasets.

The AUC is given for both the original dataset and a binarized version. Each performance is an average of 100 repetitions. In most cases the linear filter is better than SVD. The performance of the latter deteriorates quickly with an increasing number of false negatives. The performance of the linear filter remains relatively high, even with 90% of false negatives.

## References

[b1] MacKay D. J. Information Theory, Inference and Learning Algorithms (Cambridge University Press, 2003).

[b2] Gonzalez R. C. & Woods R. E. Digital Image Processing (Pearson, 2007).

[b3] Bascompte J., Jordano P., Melián C. J. & Olesen J. M. The nested assembly of plant-animal mutualistic networks. Proceedings of the National Academy of Sciences of the United States of America 100, 9383–9387 (2003). 12881488 10.1073/pnas.1633576100 PMC170927

[b4] Bastolla U. . The architecture of mutualistic networks minimizes competition and increases biodiversity. Nature 458, 1018–1020 (2009). 19396144 10.1038/nature07950

[b5] Olesen J. M., Bascompte J., Dupont Y. L. & Jordano P. The modularity of pollination networks. Proceedings of the National Academy of Sciences of the United States of America 104, 19891–19896 (2007). 18056808 10.1073/pnas.0706375104 PMC2148393

[b6] Eklöf A. . The dimensionality of ecological networks. Ecology Letters 16, 577–583 (2013). 23438174 10.1111/ele.12081

[b7] Junker R. R. . Specialization on traits as basis for the niche-breadth of flower visitors and as structuring mechanism of ecological networks. Functional Ecology 27, 329–341 (2013).

[b8] Hadfield J. D., Krasnov B. R., Poulin R. & Nakagawa S. A tale of two phylogenies: comparative analyses of ecological interactions. The American naturalist 183, 174–87 (2014). 10.1086/674445 24464193

[b9] Shimizu A. . Fine-tuned bee-flower coevolutionary state hidden within multiple pollination interactions. Scientific Reports 4, 1–9 (2014). 10.1038/srep03988 PMC3913927 24496444

[b10] Ben-Hur A. & Noble W. S. Kernel methods for predicting protein-protein interactions. Bioinformatics 21, i38–46 (2005). 15961482 10.1093/bioinformatics/bti1016

[b11] Vert J.-P., Qiu J. & Noble W. S. A new pairwise kernel for biological network inference with support vector machines. BMC Bioinformatics 8, 1–10 (2007). 18269702 10.1186/1471-2105-8-S10-S8 PMC2230501

[b12] Pelossof R. . Affinity regression predicts the recognition code of nucleic acid? Binding proteins. Nature Biotechnology 33, 1242–1249 (2015). 10.1038/nbt.3343 PMC4871164 26571099

[b13] Goldwasser L. & Roughgarden J. Sampling effects and the estimation of food-web properties. Ecology 78, 41–54 (1997).

[b14] Blüthgen N. Why network analysis is often disconnected from community ecology: A critique and an ecologist’s guide. Basic and Applied Ecology 11, 185–195 (2010).

[b15] Chacoff N. P. . Evaluating sampling completeness in a desert plant-pollinator network. Journal of Animal Ecology 81, 190–200 (2012). 21815890 10.1111/j.1365-2656.2011.01883.x

[b16] Banašek-Richter C., Cattin M.-F. & Bersier L.-F. Sampling effects and the robustness of quantitative and qualitative food-web descriptors. Journal of Theoretical Biology 226, 23–32 (2004). 14637051 10.1016/s0022-5193(03)00305-9

[b17] Fründ J., McCann K. S. & Williams N. M. Sampling bias is a challenge for quantifying specialization and network structure: lessons from a quantitative niche model. Oikos 125, 502–513 (2015).

[b18] Jordano P. Sampling networks of ecological interactions. Functional Ecology 30, 1883–1893 (2016).

[b19] Wahba G. Spline Models for Observational Data (SIAM, 1990).

[b20] Zhang S., Wang W., Ford J., Makedon F. & Pearlman J. Using singular value decomposition approximation for collaborative filtering. In Proceedings of the Seventh IEEE International Conference on E-Commerce Technology (2005).

[b21] Isinkaye F., Folajimi Y. & Ojokoh B. Recommendation systems: principles, methods and evaluation. Egyptian Informatics Journal 16, 261–273 (2015).

[b22] Staniczenko P. P. P. A., Kopp J. C. J. & Allesina S. The ghost of nestedness in ecological networks. Nature Communications 4, 1391 (2013). 10.1038/ncomms2422 23340431

[b23] Wootton J. T. & Emmerson M. Measurement of interaction strength in nature. Annual Review of Ecology, Evolution, and Systematics 36, 419–444 (2005).

[b24] Berlow E. L. . Interaction strengths in food webs: issues and opportunities. Journal of Animal Ecology 73, 585–598 (2004).

[b25] Dechtiar A. O. Parasites of fish from Lake of the Woods, Ontario. Journal of Fisheries Research Board of Canada 29, 275–283 (1972).

[b26] Kakutani T., Inoue T., Kato M. & Ichihashi H. Insect-flower relationship in the campus of Kyoto University, Kyoto: An overview of the flowering phenology and the seasonal pattern of insect visits. Contribution from the Biological Laboratory, Kyoto University 27, 465–521 (1990).

[b27] Blüthgen N., Stork N. E. & Fiedler K. Bottom-up control and co-occurrence in complex communities: honeydew and nectar determine a rainforest ant mosaic. Oikos 106, 344–358 (2004).

[b28] Blüthgen N. & Fiedler K. Preferences for sugars and amino acids and their conditionality in a diverse nectar-feeding ant community. Journal of Animal Ecology 73, 155–166 (2004).

[b29] Lafferty K. D., Dobson A. P. & Kuris A. M. Parasites dominate food web links. Proceedings of the National Academy of Sciences 103, 11211–6 (2006). 10.1073/pnas.0604755103 PMC1544067 16844774

[b30] Olesen J. M. . Missing and forbidden links in mutualistic networks. Proceedings. Biological sciences/The Royal Society 278, 725–732 (2011). 10.1098/rspb.2010.1371 PMC3030842 20843845

[b31] Jorgensen W. L. The many roles of computation in drug discovery. Science 303, 1813–1818 (2004). 15031495 10.1126/science.1096361

[b32] Lü L. . Recommender systems. Physics Reports 519, 1–49 (2012).

[b33] Zeng W., Zeng A., Liu H., Shang M. & Zhou T. Uncovering the information core in recommender systems. Scientific Reports 4, 1–14 (2014). 10.1038/srep06140 PMC4139954 25142186

[b34] Mazumder R., Hastie T. & Tibshirani R. Spectral regularization algorithms for learning large incomplete matrices. Journal of Machine Learning Research 11, 2287–2322 (2010). 21552465 PMC3087301

[b35] Stock M. . Exploration and prediction of interactions between methanotrophs and heterotrophs. Research in Microbiology 164, 1045–1054 (2013). 24012541 10.1016/j.resmic.2013.08.006

[b36] Guimerà R. & Sales-Pardo M. Missing and spurious interactions and the reconstruction of complex networks. Proceedings of the National Academy of Sciences of the United States of America 106, 22073–22078 (2009). 20018705 10.1073/pnas.0908366106 PMC2799723

[b37] Rafferty N. E. & Ives A. R. Phylogenetic trait-based analyses of ecological networks. Ecology 94, 2321–2333 (2013). 24358717 10.1890/12-1948.1 PMC3874136

[b38] Morales-Castilla I., Matias M. G., Gravel D. & Araújo M. B. Inferring biotic interactions from proxies. Trends in Ecology and Evolution 30, 347–356 (2015). 25922148 10.1016/j.tree.2015.03.014

